# Subcellular mass spectrometric detection unveils hyperglycemic memory in the diabetic heart

**DOI:** 10.1111/1753-0407.70033

**Published:** 2024-11-13

**Authors:** Jiabing Zhan, Yufei Zhou, Yifan Chen, Kunying Jin, Zhaoyang Chen, Chen Chen, Huaping Li, Dao Wen Wang

**Affiliations:** ^1^ Division of Cardiology, Tongji Hospital, Tongji Medical College Huazhong University of Science and Technology Wuhan China; ^2^ Hubei Key Laboratory of Genetics and Molecular Mechanisms of Cardiological Disorders Wuhan China; ^3^ Department of Cardiology, Fujian Medical Center for Cardiovascular Diseases, Fujian Institute of Coronary Heart Disease Fujian Medical University Union Hospital Fuzhou China

**Keywords:** diabetic cardiomyopathy, hyperglycemic memory, mass spectrometry, subcellular

## Abstract

**Background:**

Intensive glycemic control is insufficient to reduce the risk of heart failure in patients with diabetes mellitus. While the hyperglycemic memory in the diabetic cardiomyopathy has been well documented, its underlying mechanisms are not fully understood. The present study tried to investigate whether the dysregulated proteins/biological pathways, which persistently altered in diabetic hearts during normoglycemia, participate in the hyperglycemic memory.

**Methods:**

Hearts of streptozotocin‐induced diabetic mice, with or without intensive glycemic control using slow‐release insulin implants, were collected. Proteins from total heart samples and subcellular fractions were assessed by mass spectrometry, Western blotting, and KEGG pathway enrichment analysis. mRNA sequencing was used to determine whether the persistently altered proteins were regulated at the transcriptional or post‐transcriptional level.

**Results:**

Western blot validation of several proteins with high pathophysiological importance, including MYH7, HMGCS2, PDK4, and BDH1, indicated that mass spectrometry was able to qualitatively, but not quantitatively, reflect the fold changes of certain proteins in diabetes. Pathway analysis revealed that the peroxisome, PPAR pathway, and fatty acid metabolism could be efficiently rescued by glycemic control. However, dysregulation of oxidative phosphorylation and reactive oxygen species persisted even after normalization of hyperglycemia. Notably, mRNA sequencing revealed that dysregulated proteins in the oxidative phosphorylation pathway were not accompanied by coordinated changes in mRNA levels, indicating post‐transcriptional regulation. Moreover, literature review and bioinformatics analysis suggested that hyperglycemia‐induced persistent alterations of miRNAs targeted genes from the persistently dysregulated oxidative phosphorylation pathway, whereas, oxidative phosphorylation dysfunction‐induced ROS regulated miRNA expression, which thereby might sustained the dysregulation of miRNAs.

**Conclusions:**

Glycemic control cannot rescue hyperglycemia‐induced alterations of subcellular proteins in the diabetic heart, and persistently altered proteins are involved in multiple functional pathways, including oxidative phosphorylation. These findings might provide novel insights into hyperglycemic memory in diabetic cardiomyopathy.

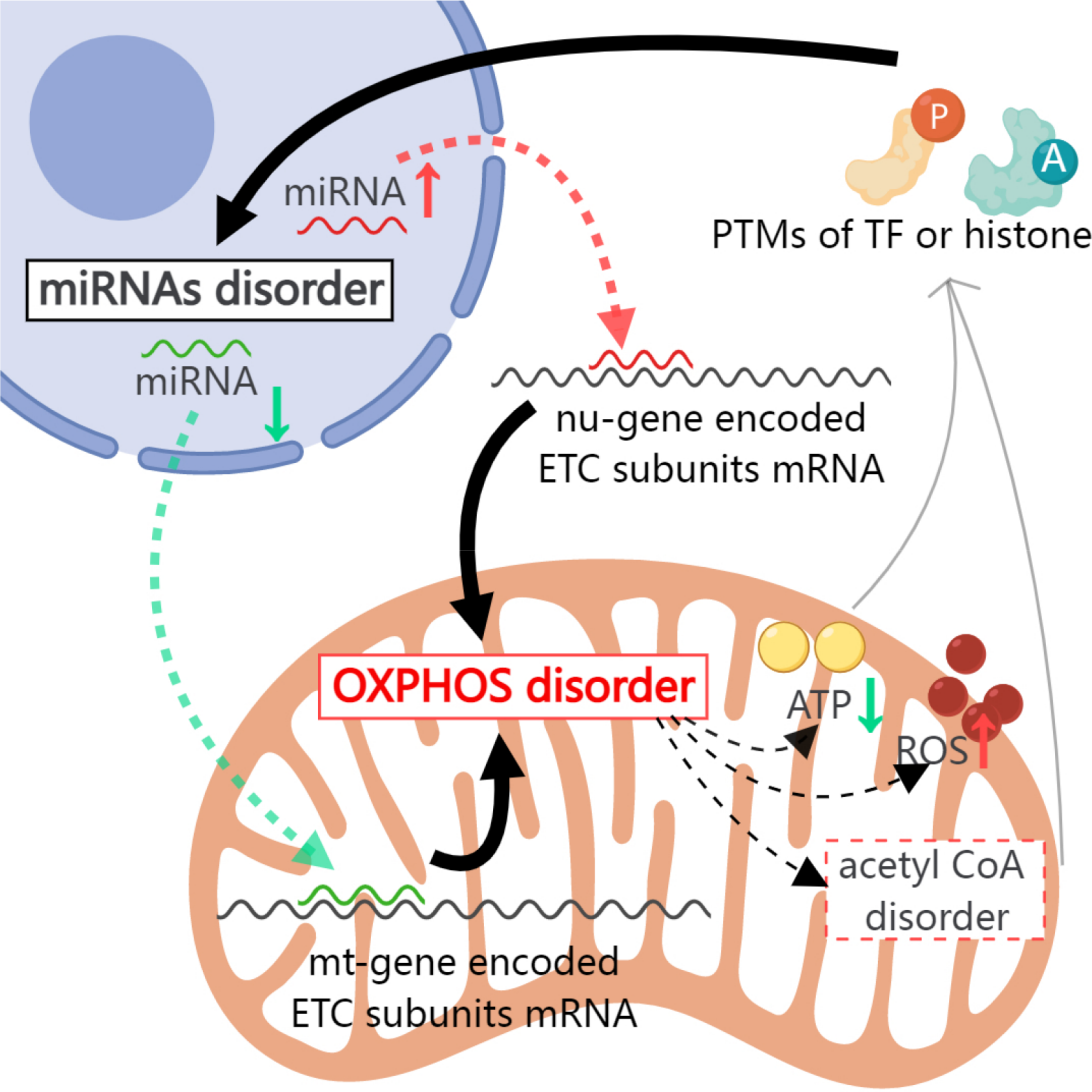

## Graphical abstract

**Highlights**

- Our results suggested that mass spectrometry was qualitatively, but not quantitatively, reflect the fold changes of proteins in diabetes.
- We revealed that glucose control was unable to rescue hyperglycemia‐induced alterations of subcellular proteins in the diabetic heart.
- A potential vicious cycle between dysregulated miRNAs and disordered oxidative phosphorylation provides novel insights into hyperglycemic memory in diabetic cardiomyopathy.

## 1 BACKGROUND

The global prevalence of diabetes in 2019 was estimated to be 9.3% (463 million people) and is predicted to rise to 10.2% (578 million) by 2030 and 10.9% (700 million) by 2045.^1^ Cardiovascular complications account for nearly 80% of deaths due to diabetes complications.^2^ The concept of diabetic cardiomyopathy was proposed as early as 1974 and is defined as the appearance of abnormal myocardial structure and performance in the absence of hypertension, coronary heart disease, severe valvular disease, and other conventional cardiovascular risk factors in individuals with diabetes.^3^ Glycemic control has long been considered one of the most important therapeutic approaches for preventing diabetes‐related complications. However, multiple recent large‐scale studies have revealed that SGLT2i‐containg glycemic control therapy delays disease progression.^4^, ^5^, ^6^, ^7^ The anti‐hyperglycemia drugs that have the ability to halt or even reverse disease progression remain to be discovered. These studies suggested that patients with diabetes are prone to cardiovascular complications even after intensive blood glucose control, a condition that is now referred to as “hyperglycemic memory” (HGM) phenomenon.^8^, ^9^

Multiple molecular pathways, such as advanced glycation end products (AGEs), oxidative stress, miRNAs, and epigenetic modifications, have been suggested to participate in this phenomenon.^10^ Glycemic control did not rescue hyperglycemia‐induced alterations in the miRNA landscape in the diabetic heart.^11^ Our previous studies have also shown that miR‐320 promotes CD36 expression, which in turn, was able to increase miR‐320 expression. This vicious cycle leads to sustained elevation of free fatty acid (FFA) uptake and myocardial lipotoxicity, even after normalization of hyperglycemia.^12^ However, these studies revealed only isolated proteins involved in HGM, while global alterations in the protein landscape are not fully understood. Moreover, HGM‐related miRNAs appear to play distinct roles in different subcellular fractions^12^, ^13^, ^14^; in contrast, alterations in the protein landscape in the subcellular fractions are unknown.

In the current study, we used mass spectrometry (MS) to determine whether proteins localized in different subcellular fractions could be rescued by glycemic control in the diabetic heart. Here, we show that MS results can qualitatively represent changes in proteins. Hyperglycemia significantly affects subcellular protein expression in the hearts of diabetic mice, and these detrimental signatures are retained despite intensive glycemic control.

## 2 METHODS

### 2.1 STZ treatment

Streptozotocin (STZ; Sigma, Shanghai, China) was dissolved in a sodium citrate buffer (pH 4.5) to a concentration of 8 mg/mL. C57BL/6J mice (8‐week‐old males) were deprived of food overnight and injected intraperitoneally with 40 mg/kg STZ, which was conducted for consecutive 5 days. Control mice were injected with an equivalent volume of sodium citrate buffer.^11^ All the experimental mice were measured for their blood glucose levels, with a range of ≥250 mg/dL considered diabetic.

### 2.2 Insulin pump implantation

**FIGURE 1:**
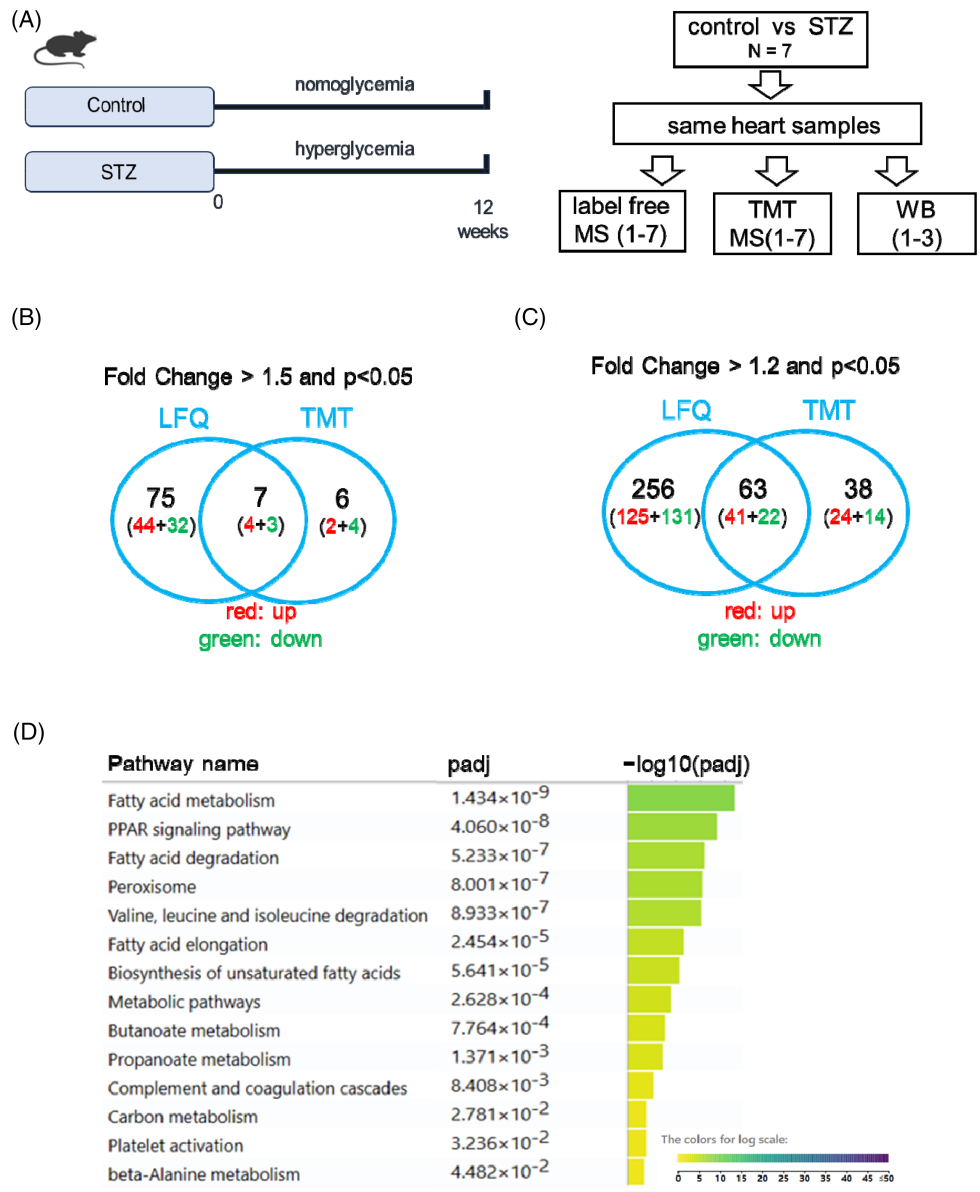
Comparison between label‐free and TMT‐based mass spectrometry analysis on total heart samples from STZ mice. (A) Experiment design of three protein quantification strategies in diabetic heart. STZ, streptozotocin; TMT, tandem mass Tag; WB, Western blot. (B) Venn diagrams showing the numbers and overlap of dysregulated proteins in hearts (control versus STZ) between LFQ and TMT‐based mass spectrometry (MS) analysis, using the cutoff *p* < 0.05 and fold change >1.5. (C) Venn diagrams showing the numbers and overlap of dysregulated proteins in hearts (control versus STZ) between LFQ and TMT‐based MS analysis, using the cutoff *p* < 0.05 and fold change >1.2. (D) The KEGG pathway enrichment analysis of 63 proteins which dysregulated in both LFQ and TMT‐based MS analysis. *p* < 0.05 and fold change >1.2.

Alzet Mini‐Osmotic Pump 1004 (Durect, Cupertino, CA, USA) was used for insulin supplement according to the manufacturer's protocol. Briefly, right before implantation, the osmotic pumps were infused with 100 μL insulin aspart injection (100 U/mL, NovoRapid FlexPen, Novo Nordisk) and incubated at 37°C for 48 h, which would then achieve a stable insulin release rate of 0.11 μL/h. Four weeks after the induction of diabetes, the mice in the blood glycemic control group were anesthetized with 1% pentobarbital and implanted with the prepared osmotic pump for another 4 weeks. Detailed information on the animal studies is shown in the Figures 1A and 4A.

### 2.3 Subcellular fractionation

Cytoplasmic and nuclear fractions were prepared using a Nuclear‐Cytosol Extraction Kit (APPLYGEN, Beijing, China) according to the manufacturer's instructions. Specifically, the tissue homogenate was centrifuged at 12 000×*g* for 5 min at 4°C and the supernatant was transferred to a new tube as the cytosolic fraction. The pellet was purified and collected as the nuclear fraction. The mitochondrial fraction was isolated from the mouse heart using the MACS Mitochondria Extraction Kit (Miltenyi Biotec GmbH, Bergisch Gladbach, Germany) according to the manufacturer's recommendations. Briefly, pre‐weighed mouse hearts were gently homogenized, and mitochondria in the homogenate were magnetically labeled using Tomm20‐coated beads, followed by magnetic separation. Eluted mitochondria were then collected by centrifugation at 13 000×*g* for 2 min at 4°C. The non‐cytosolic fraction was a mixture of nuclei and mitochondria isolated from the mouse hearts.

### 2.4 Proteomics

Quantitative proteomics was conducted and analyzed using APTBIO (Shanghai, China).

Protein extraction and digestion: For both label‐free and TMT samples, SDT buffer (4% SDS, 100 mM Tris–HCl, pH 7.6) was used for mouse heart lysis and protein extraction. The amount of protein was quantified using a BCA Protein Assay Kit (Bio‐Rad, USA). The proteins were digested with trypsin according to the filter‐aided sample preparation (FASP) procedure described by Mann. The digest peptides were desalted on C18 Cartridges (Empore™ SPE Cartridges C18 (standard density), bed I.D. 7 mm, volume 3 mL, Sigma), concentrated by vacuum centrifugation and reconstituted in 40 μL of 0.1% (v/v) formic acid.

TMT labeling: Processed peptides (100 μg) per sample were labeled using TMT reagent according to the manufacturer's instructions (Thermo Scientific). The labeled peptides were fractionated using a high pH Reversed‐Phase Peptide Fractionation Kit (Thermo Scientific). The collected fractions were desalted on C18 Cartridges (Empore SPE Cartridges C18 (standard density), bed I.D. 7 mm, volume 3 mL; Sigma) and concentrated by vacuum centrifugation.

LC‐MS/MS analysis: For both label‐free and TMT quantification strategies, LC‐MS/MS analysis was performed using a Q Exactive mass spectrometer (Thermo Fisher Scientific) coupled to an Easy nLC (Proxeon Biosystems, now Thermo Fisher Scientific). The mass spectrometer was operated in the positive ion mode.

Identification and quantification of proteins: The label‐free raw MS data for each sample were combined and searched using MaxQuant 1.6.14 software for identification and quantitation analysis. The TMT‐MS raw data for each sample were searched using the MASCOT engine (Matrix Science, London, UK; version 2.2) embedded into Proteome Discoverer 1.4 software for identification and quantitation analysis.

### 2.5 Western blotting

Protein concentrations were determined using Bradford assay. Lysates were resolved using 10% (wt/vol) acrylamide gel SDS‐PAGE electrophoresis, followed by transfer to a polyvinylidene fluoride (PVDF) membrane. Bovine serum albumin (5%) was used to block nonspecific sites for 2 h at room temperature. The membrane was then incubated with indicated primary antibody overnight at 4°C, followed by peroxidase‐conjugated secondary antibody for 2 h at room temperature. Protein bands were visualized using an enhanced chemiluminescence kit. Results from Western blotting were quantified by densitometry and processed using ImageJ software (National Institutes of Health software). The antibodies used in this study are listed in Table S1.

### 2.6 mRNA‐seq

RNA was extracted from the hearts of control and STZ‐treated mice using TRIzol reagent (Invitrogen Life Technologies). RNA reverse transcription, library construction, and sequencing were performed by Majorbio Bio‐Pharm Biotechnology (Shanghai, China).

### 2.7 Histological analysis

Dihydroethidium (DHE; Invitrogen, Carlsbad, CA) was applied to 7 μm frozen heart slices at 10 μmol/L for 20 min to detect oxidative stress levels of the hearts. Fluorescent images were acquired using a Carl Zeiss fluorescence microscope (AXIO IMAGER A2), and the fluorescence intensity was analyzed using ImageJ software.

### 2.8 Bioinformatics

Bioinformatic prediction of potential miRNAs targeting nuclear‐encoded transcripts was performed using TargetScan software, whereas miRNAs targeting mitochondrial gene‐encoded transcripts were predicted using RNAhybrid software. KEGG pathway analysis was performed using g:Profiler, a web server for functional enrichment analysis and conversion of gene lists.^15^

### 2.9 Statistical analysis

Data are presented as the mean ± standard error of the mean (SEM); n is noted in specific figure legends. All data set were tested for normality using the Shapiro–Wilk test in order to select an appropriate parametric or non‐parametric test. Student's *t* tests were performed to determine statistical significance between two groups. In all cases, statistical significance was defined as *p* < 0.05.

## 3 RESULTS

### 3.1 Comparison between label free and TMT‐based mass spectrometry analysis on total heart samples from STZ mice

To obtain a global expression pattern of dysregulated proteins in the STZ diabetic hearts, we performed label‐free (LFQ) and tandem mass tag (TMT)‐based mass spectrometry analyses on total hearts of mice 12 weeks after STZ treatment. The glycemic levels and cardiac function were assessed to confirm the induction of diabetes and the comprised cardiac diastolic function, as showed in our previous study.^12^ Using these samples, we performed mass spectrometric analysis using the LFQ and TMT methods to evaluate the regulatory trend of proteins in STZ mouse hearts (Figure 1A).

Using LFQ, 2412 proteins were detected in the mouse heart. Using a cutoff fold‐change value of 1.5 and *p* < 0.05 (*t* test), 48 proteins were upregulated, while 35 proteins were downregulated in the STZ heart (Excel S1). When the cutoff fold‐change value was 1.2 and *p* < 0.05, 166 proteins were upregulated and 153 proteins were downregulated (Excel S1). In contrast, using the TMT method, 3011 proteins were detected in the heart, which far exceeded the number of proteins detected using the LFQ method. However, using a cutoff fold change value of 1.5 and *p* < 0.05 (*t* test), only six proteins were upregulated, while seven proteins were downregulated in the STZ heart. When the cutoff fold change value of 1.2 and *p* < 0.05 were used, 65 proteins were upregulated while 36 proteins were downregulated (Excel S2). Comparison between the LFQ and TMT methods showed that seven proteins were consistently dysregulated in the STZ heart using a cutoff fold change value of 1.5, whereas 63 proteins were dysregulated when using a cutoff fold change value of 1.2 (Figure 1B,C). Pathway analysis of the 63 dysregulated proteins revealed that fatty acid metabolism, PPAR signaling pathway, and fatty acid degradation were the top pathways dysregulated in the STZ group (Figure 1D). Previous studies have reported that these pathways play critical roles in diabetic cardiomyopathy.^16^, ^17^, ^18^, ^19^, ^20^, ^21^

### 3.2 WB validation of the mass spectrometry in STZ heart

**FIGURE 2:**
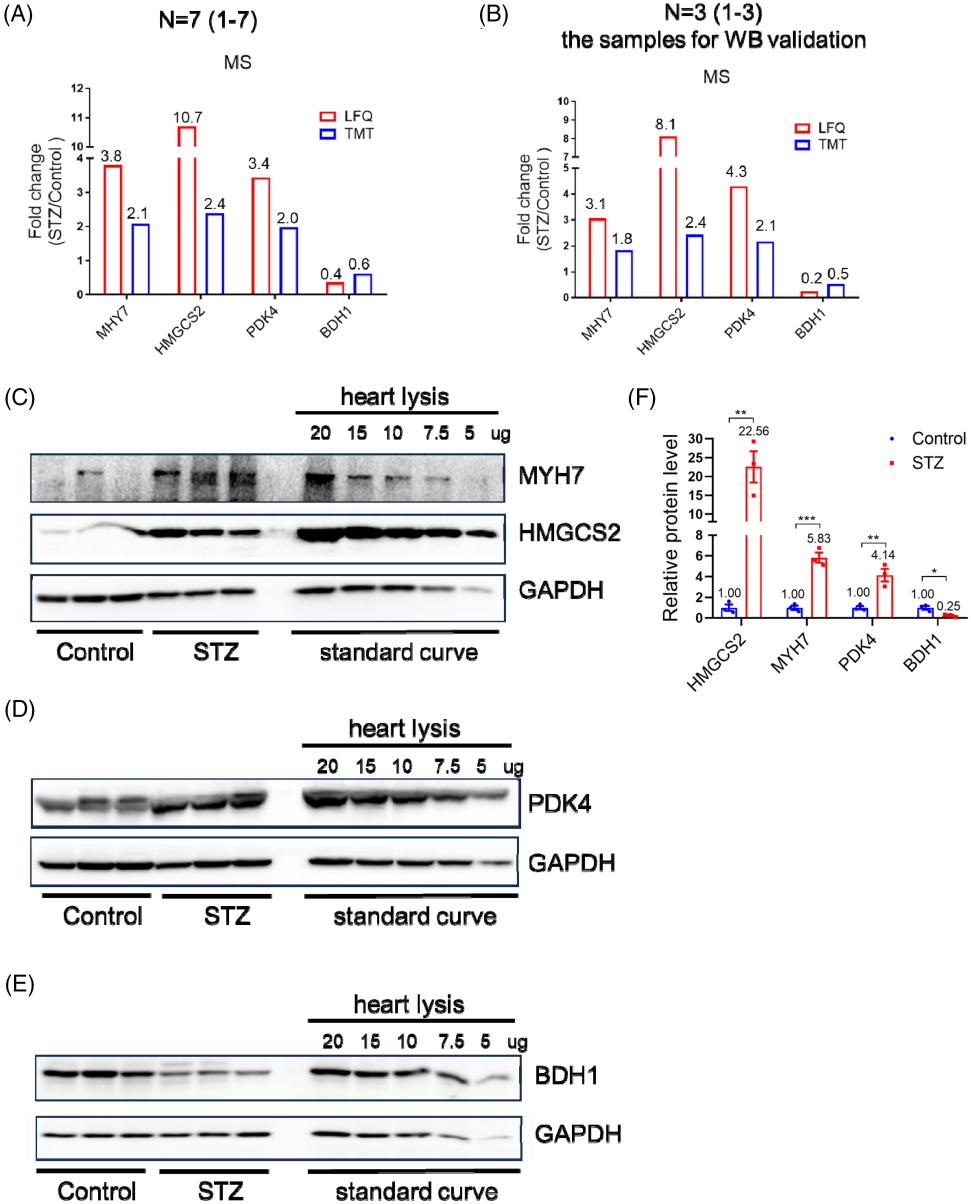
WB validation of the mass spectrometry in STZ heart. (A) The protein expression of MYH7, HMGSC2, PDK4, BDH1 revealed by MS in control and STZ‐treated mice hearts. Data was presented as fold change. *N* = 7. (B) The protein expression of MYH7, HMGSC2, PDK4, BDH1 revealed by MS in control and STZ‐treated mice hearts used for subsequent WB validation. Data was presented as fold change. *N* = 3. (C–E) Western blot analysis of MYH7, HMGSC2, PDK4, BDH1 protein levels in STZ‐treated and control mice hearts. Quantified heart lysis protein was simultaneously loaded in the same gel as standard curve. (F) Quantification analysis of WB in C, D, and E. *N* = 3. Student's *t* test. **p < 0.01, ***p < 0.001.

Using MS analysis, we identified 63 dysregulated proteins, including 41 upregulated and 22 downregulated proteins. To test whether MS analysis represented real fold changes in dysregulated proteins, we performed Western blot validation of dysregulated proteins. Specifically, several proteins with very high pathophysiological importance, such as MYH7, HMGCS2, PDK4, and BDH1^22^, ^23^, ^24^, ^25^, ^26^ were selected for Western blot validation, with diluted proteins serving as standard curves. Relative to the fold changes in these proteins revealed by MS (Figure 2A,B), Western blotting showed much higher fold changes for HMGCS2 in the STZ group (Figure 2C–F). These data suggested that MS was able to qualitatively, but not quantitatively, represent the fold changes of certain dysregulated proteins during diseases compared to Western blotting.

### 3.3 TMT‐based mass spectrometry analysis on subcellular localized protein from STZ heart

The consistent regulatory trend between MS and Western blot validation of critical proteins suggested that MS is a conceivable method to investigate protein changes during diseases (although it may not reflect the real fold changes). We next used MS to evaluate the expression pattern of subcellular localized proteins in STZ‐treated hearts. Because the TMT method can detect many more proteins, which indicates higher sensitivity, it was used for subsequent MS experiments on subcellular‐localized proteins. In contrast, the number of proteins detected in the heart was reduced using the LFQ method. Even proteins that were detectable in most samples were not detected in specific heart samples. For example, Hp, which was the most downregulated protein, was only detectable in 11 of the 14 hearts using the LFQ method. Therefore, the TMT method was more reliable than LFQ in determining the expression levels of proteins and was used in subsequent studies.

**FIGURE 3:**
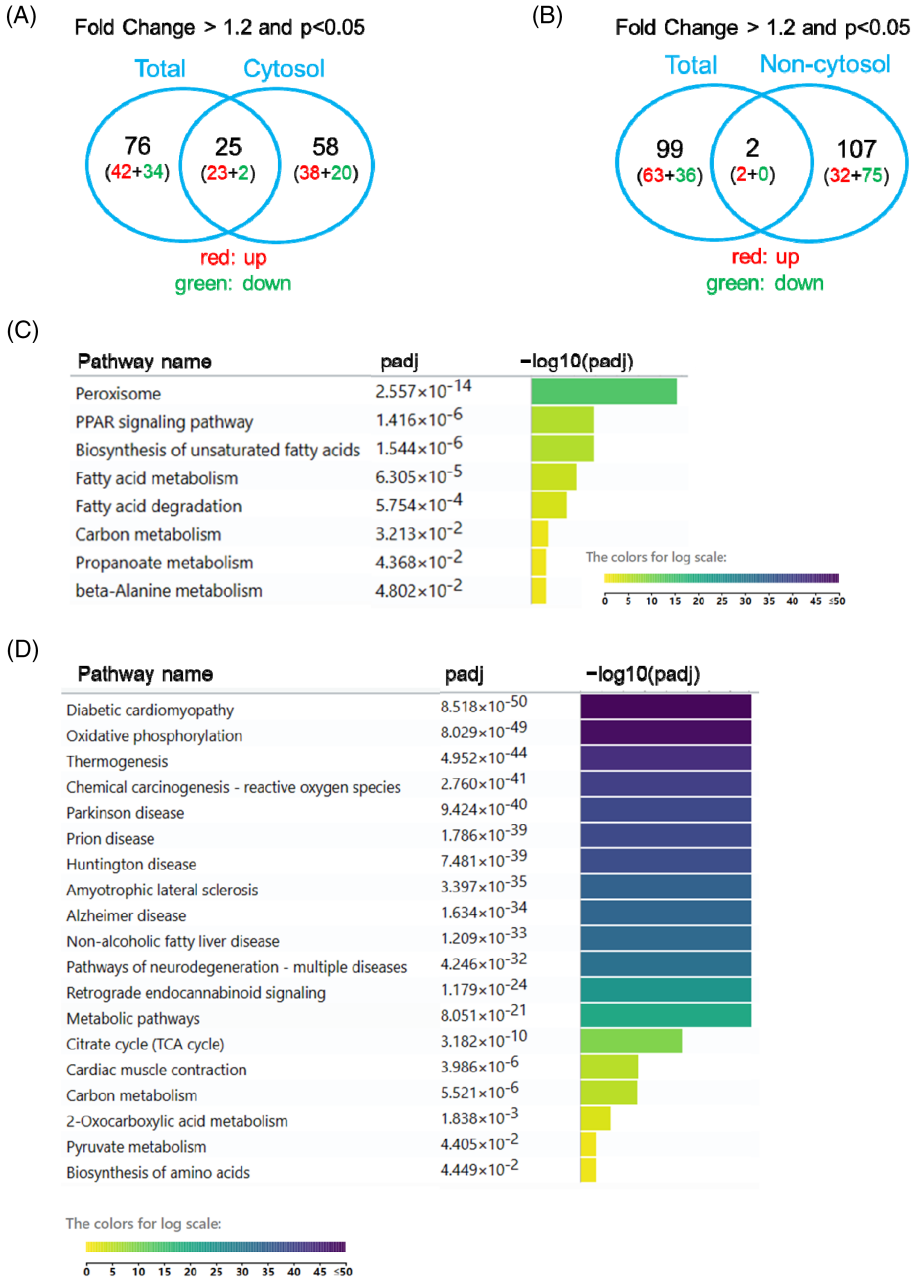
TMT‐based mass spectrometry analysis on subcellular localized protein from STZ heart. (A) Venn diagrams showing the numbers and overlap of dysregulated proteins of mice (control versus STZ) between total and cytosol heart fraction, using the cutoff *p* < 0.05 and fold change> 1.2. (B) Venn diagrams showing the numbers and overlap of dysregulated proteins of mice (control versus STZ) between total and non‐cytosol heart fraction, using the cutoff *p* < 0.05 and fold change> 1.2. (C) The KEGG pathway enrichment analysis of 83 proteins dysregulated in cytosol fraction of STZ‐treated mouse heart compared with their controls. *p* < 0.05 and fold change> 1.2. (D) The KEGG pathway enrichment analysis of 109 proteins dysregulated in non‐cytosol fraction of STZ‐treated mouse heart compared with their controls. *p* < 0.05 and fold change> 1.2.

Control and STZ‐treated mouse hearts were subjected to isolation of subcellular fractions 8 weeks after STZ treatment. Subsequently, the cytosolic and non‐cytosolic fractions (nucleus and mitochondria) were used for MS analysis. Notably, in the cytosolic fraction, 83 proteins were dysregulated, of which 61 were upregulated and 22 were downregulated in the STZ‐treated hearts (Excel S3). Of the 83 proteins, only 25 showed a consistent regulatory pattern throughout the heart (Figure 3A). Fifty‐eight proteins showed upregulation or downregulation change only in cytosol but not in total heart, which might be due to the following reason: these proteins have multiple localization characteristics and display distinct regulation patterns in different subcellular fractions. Therefore, their expression patterns in the subcellular fractions may be very different from those in the total heart.

In terms of protein expression in the non‐cytosolic fractions, 34 proteins were upregulated and 75 were downregulated, resulting in 109 dysregulated proteins (Excel S4). Comparison between total MS and MS in the non‐cytosolic fraction revealed that only two proteins were consistently dysregulated (Figure 3B), indicating that the changes in subcellular fractions were very different from those of the total heart in STZ‐treated mice.

Pathway analyses of cytosolic (83 proteins) and non‐cytosolic dysregulated proteins (109 proteins) revealed distinct patterns. Specifically, in the cytosolic fractions, the peroxisome, PPAR signaling pathway, and unsaturated fatty acid biosynthesis were the top pathways, while in the non‐cytosolic fractions, diabetic cardiomyopathy and oxidative phosphorylation pathways were the top pathways (Figure 3C,D).

### 3.4 TMT based mass spectrometry analysis on subcellular localized protein from STZ heart with glycemic control

**FIGURE 4:**
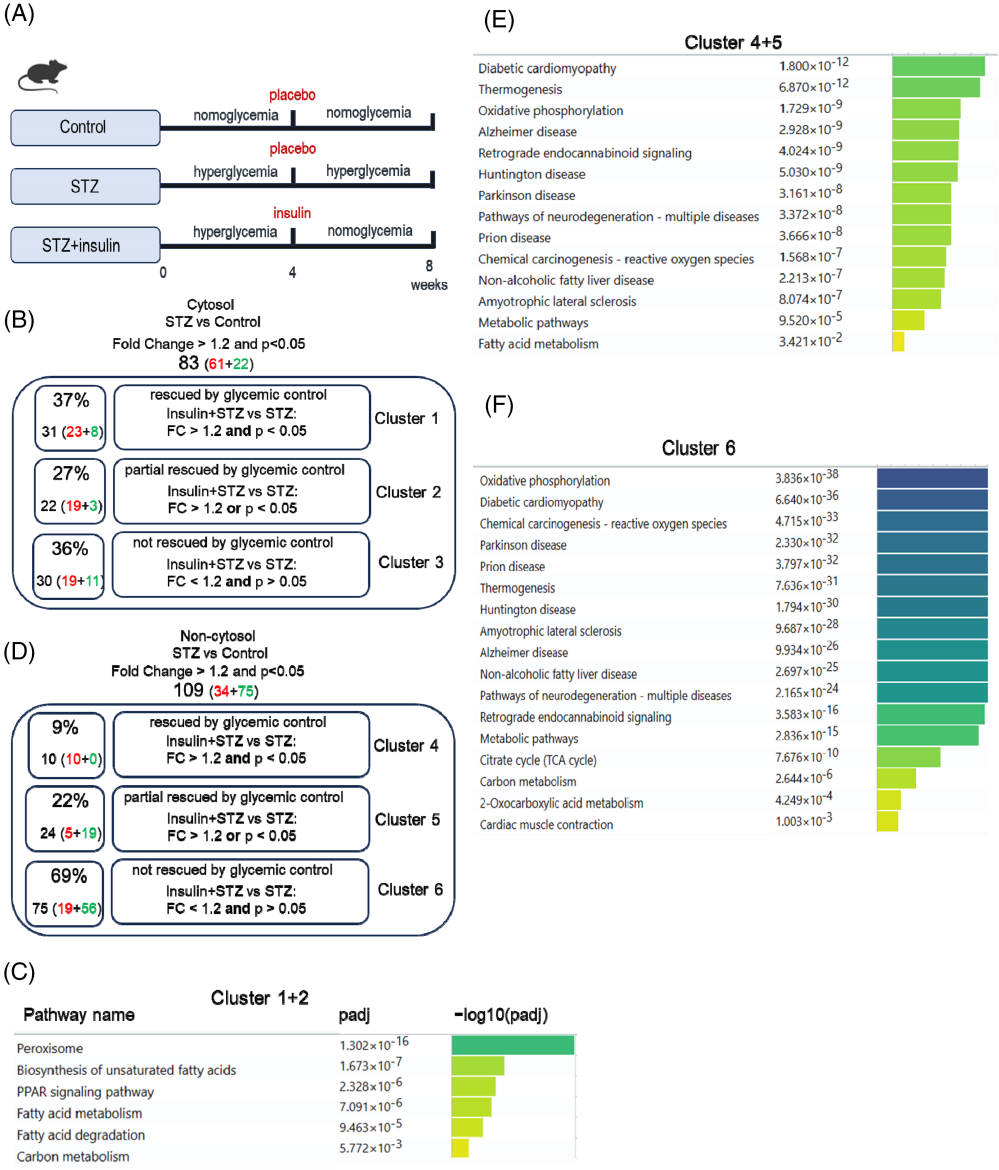
TMT‐based mass spectrometry analysis on subcellular localized protein from STZ heart with glycemic control. (A) Schematic of animal experiment design. Glycemic control was achieved by insulin after 4 weeks of hyperglycemia in STZ‐treated mice. (B) Dysregulated proteins in cytosol fraction of hearts (from controls, diabetic mice, and diabetic animals treated with insulin) were divided into three clusters (Clusters 1, 2, and 3) according to their regulating pattern. *p* < 0.05 and fold change> 1.2. (C) The KEGG pathway enrichment analysis of dysregulated cytosol proteins in Clusters 1 and 2, which could be rescued to some extent by glycemic control in diabetic mice. *p* < 0.05 and fold change> 1.2. (D) Dysregulated proteins in non‐cytosol fraction of hearts (from controls, diabetic mice and diabetic animals treated with insulin) were divided into three clusters (Clusters 4, 5, and 6) according to their regulating pattern. *p* < 0.05 and fold change> 1.2. (E) The KEGG pathway enrichment analysis of dysregulated non‐cytosol proteins in cluster 4 and cluster 5, which could be rescued to some extent by glycemic control in diabetic mice. *p* < 0.05 and fold change> 1.2. (F) The KEGG pathway enrichment analysis of dysregulated non‐cytosol proteins in cluster 6, which failed to be rescued by glycemic control in diabetic mice. *p* < 0.05 and fold change> 1.2.

Following the identification of distinct regulation pattern of cytosolic and non‐cytosolic localized proteins in STZ‐treated mice, we tested whether glycemia control by insulin could rescue these significantly dysregulated proteins/pathways. Our previous study revealed that, after 4 weeks of hyperglycemic stress, insulin treatment was effective in controlling hyperglycemia, but was no longer able to prevent or reverse cardiac diastolic dysfunction, indicating that hyperglycemic memory was established 4 weeks after hyperglycemic stress.^12^ In this study, hearts from diabetic mice, which received 4 weeks of insulin treatment after 4 weeks of hyperglycemia (Figure 4A), were subjected to isolation of subcellular fractions. TMT‐based mass spectrometry analysis was then used to evaluate the expression of subcellular proteins in insulin‐treated STZ mice.

In the cytosolic fractions, the 83 dysregulated proteins in the STZ hearts were divided into three clusters (Figure 4B). Cluster 1 contained 31 proteins (37%) that were significantly rescued by glycemic control (insulin treatment) with a fold change cutoff value >1.2 and *p* value <0.05. Cluster 2 contained 22 proteins (27%) that could be partially rescued, which we defined as having a fold‐change cutoff value >1.2 and *p* value <0.05. Cluster 3 contained 30 proteins (36%) that could not be rescued by glycemic control (Figure 4B, Excel S5). Pathway analysis of combined proteins from clusters 1 and 2 revealed that peroxisomes, fatty acid biosynthesis, PPAR signaling pathway, fatty acid metabolism, and degradation were rescued to some extent by glycemic control (Figure 4C). However, for the 30 proteins in cluster 3, which likely represent a characteristic of hyperglycemic memory, no significant pathways were found (Excel S5). Interestingly, among the 30 persistently altered proteins, CD36 was successfully validated and systemically evaluated in our previous studies.^12^, ^27^ Therefore, the MS data used to evaluate the persistently altered proteins in STZ were convincing.

In the non‐cytosolic fractions (mainly nucleus and mitochondria), 109 proteins were dysregulated in STZ mouse hearts, of which 10 (9%) proteins were significantly rescued, 24 (22%) were partially rescued, and 75 (69%) could not be rescued (Figure 4D, Excel S6). Pathway analysis revealed that the rescued genes (Clusters 4 and 5) were involved in diabetic cardiomyopathy, thermogenesis, and oxidative phosphorylation pathways (Figure 4E). For the large number of unreversed proteins (Cluster 6), they were involved in the oxidative phosphorylation, diabetic cardiomyopathy, and reactive oxygen species pathways (Figure 4F). These data revealed that dysregulation of the oxidative phosphorylation pathway (the Top 1 pathway) was still present even after normalization of hyperglycemia, indicating its hyperglycemic memory nature.

### 3.5 Potential feedback loop between persistently dysregulated oxidative phosphorylation pathway and miRNAs

**FIGURE 5:**
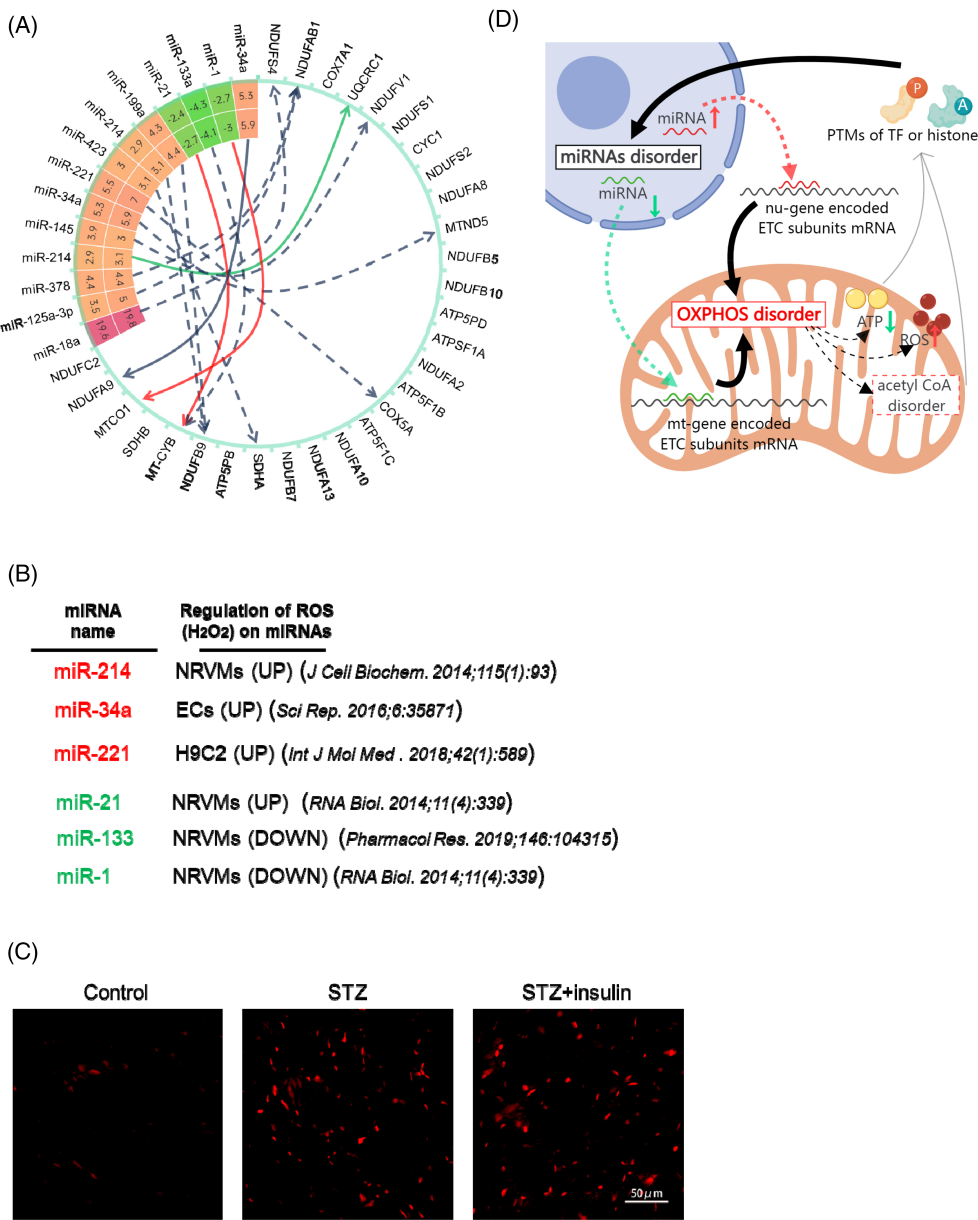
Potential feedback loop between persistently dysregulated oxidative phosphorylation pathway and miRNAs. (A) miRNAs having hyperglycemic memory potentially target genes in oxidative phosphorylation pathway. Inner ring: dysregulating fold changes (STZ vs. control) of miRNAs; outer ring: dysregulating fold changes (STZ + insulin vs. control) of miRNAs; red solid arrow: validated upregulation of miRNA on target gene in previous studies; green solid arrow: validated downregulation of target gene by miRNA in previous studies; dotted gray arrow: predicted regulation of miRNA on target gene. (B) Some miRNAs having hyperglycemic memory were reported to be regulated by H_2_O_2_. Left row: MiRNA name; right row: cell types and regulating pattern of H_2_O_2_ on specific miRNAs in previous studies. (C) Images of DHE staining of cardiac sections in different groups (control, STZ‐induced diabetic mice, STZ‐induced diabetic mice treated with insulin). (D) Schematic illustrating the hypothetical of a feedback loop which contains dysregulated miRNAs and disordered mitochondrial oxidative phosphorylation. ETC, electron transport chain; OXPHO, oxidative phosphorylation; PTMs, post‐translation modifications; ROS, reactive oxygen species; TF, transcription factors.

Results stated in previous sections demonstrate the persistence of the dysregulated oxidative phosphorylation pathway even after glycemic control. To investigate whether dysregulation at the protein level was secondary to changes in mRNA levels, we performed mRNA sequencing of the hearts of STZ mice. Notably, the mRNA levels of these persistently dysregulated genes remained unchanged (Excel S7), indicating that these proteins in the oxidative phosphorylation pathway are regulated at the post‐transcriptional level. One of the most studied mechanisms of post‐transcriptional regulation is miRNA‐mediated translational control.^28^, ^29^ There is likely a close link between dysregulated miRNAs and persistently altered oxidative phosphorylation dysfunction. A previous study demonstrated that dysregulation of 316 miRNAs was observed in the STZ diabetic hearts compared with controls. Of these, the expression of 268 miRNAs remained significantly altered in diabetic mice, even after subsequent normoglycemia.^11^ Comparison information about glycemic control in these two studies was showed in Table S2. Using TargetScan and RNAhybrid software, we searched for potential miRNAs that may target the 3′UTR of nu‐gene encoded transcripts or CDS region of mt‐gene encoded transcripts (mitochondrial mRNAs lack 3′UTR) that were involved in the persistently dysregulated oxidative phosphorylation pathway. We found that several miRNAs (miR‐18a, miR‐125a‐3p, miR‐378, miR‐214, miR‐145, miR‐34a, miR‐221, miR‐423, miR‐199a, miR‐21, miR‐133, and miR‐1) that remained significantly altered in diabetic mice even after normoglycemia were predicted to target oxidative phosphorylation‐related genes (Figure 5A). Most miRNAs negatively regulate gene expression through translational inhibition or mRNA cleavage.^30^ The persistent upregulation of miRNAs (such as miR‐18a) may be responsible for the downregulation of oxidative phosphorylation‐related genes (such as NDUFS4). However, miRNAs localized in the mitochondria are upregulated rather than suppressed during mt‐genes translation.^31^ Therefore, the persistent downregulation of miRNAs such as miR‐21, miR‐133, and miR‐1 may be responsible for the continuous downregulation of mt‐subunits such as mt‐CYTB and mt‐CO1 in the oxidative phosphorylation pathway. Examples of the inhibitory effect of miRNAs on nu‐gene‐encoded electron transfer chain (ETC) subunits and the up‐regulatory effect on mt‐gene‐encoded ETC subunits have been reported in previous studies.^32^ We propose a model in which persistent disorders of miRNAs may be the leading cause of oxidative phosphorylation dysfunction.

We then investigated the reason for persistence of hyperglycemia‐induced alterations in the miRNA landscape in the STZ heart even after normoglycemia. Many studies have demonstrated that H_2_O_2_ can regulate miRNA expression in cardiomyocytes, including miRNAs with hyperglycemic memory, such as miR‐214, miR‐221, and miR‐1 (Figure 5B).^33^, ^34^, ^35^, ^36^ Interestingly, we also observed a clear upregulation of reactive oxygen species (ROS) in the STZ diabetic heart, which was not rescued by glycemic control (Figure 5C). Therefore, we propose a potential feedback loop involving dysregulated miRNAs and disordered mitochondrial oxidative phosphorylation. Once established, this feedback loop persisted even after the normalization of hyperglycemia in the diabetic model (Figure 5D). Although this hypothesis needed further tested, and the starting point and detailed mechanisms underlying this loop remain unclear and require further investigation, the proposed feedback loop theory provides new insights into the hyperglycemic memory phenomenon in diabetes‐induced cardiac dysfunction.

## 4 DISCUSSION

The present study, for the first time, suggests that diabetes induces a profound alteration in protein expression in the subcellular fractions of the heart and, most importantly, that these detrimental signatures are not reversed by glycemic control. Specifically, a persistent alteration in oxidative phosphorylation‐related proteins in non‐cytosolic fractions suggests the existence of a hyperglycemic memory in the heart. Previous studies^10^, ^11^, ^12^ suggest that some hyperglycemic memory‐related miRNAs are able to regulate the expression of genes involved in oxidative phosphorylation, whereas oxidative phosphorylation dysfunction‐induced ROS (such as H_2_O_2_) upregulation is able to modify miRNA expression. This potential feedback loop between disordered miRNAs and mitochondrial oxidative phosphorylation provides a new explanation for persistent cardiac dysfunction even after glycemic control, which needed further confirmation.

In this study, MS was used to detect a large number of proteins in an unbiased manner. However, MS technology is not inherently quantitative because the peptide response depends on the sample matrix and ionization efficiencies, which are nonlinear responses in many cases.^37^, ^38^, ^39^ By comparing the LFQ‐ and TMT‐based MS results, the TMT method appeared to be more stable and reliable, as it could detect far more proteins than the LFQ method. Moreover, some high‐abundance proteins were undetectable in several heart samples by LFQ but could be steadily detected in all heart samples by TMT. Therefore, TMT was selected over LFQ to study the subcellular proteins under STZ treatment and glycemic control conditions. Moreover, we found that the fold changes of specific proteins revealed by MS were not as dramatic as the Western blot results using the standard curve, indicating that MS can qualitatively, but not quantitatively, determine changes in certain proteins in diseases. Although the fold‐changes of TMT‐based MS were condensed compared to the western blot data, their regulation trends were consistent. To date, MS remains the only approach for obtaining unbiased global protein expression data.

Using TMT‐based MS, we observed many dysregulated pathways in the STZ heart, such as fatty acid metabolism, the PPAR signaling pathway, fatty acid degradation, peroxisomes, oxidative phosphorylation, and reactive oxygen species. These pathways have been extensively studied before.^16^, ^17^, ^18^, ^19^, ^20^, ^21^ Notably, several pathways, such as oxidative phosphorylation and reactive oxygen species, were dysregulated in the non‐cytosolic fraction, but not in the total heart lysis by MS detection. This discrepancy might be due to (1) some mt‐subunits displaying distinct regulation patterns in different subcellular fractions; for example, Uqcrc1 and Cox7a1 from the oxidation phosphorylation pathway were significantly decreased in the non‐cytosolic fraction (STZ vs. control:0.74, Uqcrc1 and 0.73 for Cox7a1) while showing an upregulation trend in the cytosol (STZ vs. control:1.03, Uqcrc1 and 1.05 for Cox7a1). (2) Presence of abundant cytosol proteins might interfere with the detection efficiency of mitochondrial proteins by MS. Take mitochondrial gene encoded mt‐Cytb for example, in non‐cytosol fraction the fold change was 0.82 (STZ vs. Control), but in total heart the fold change was only 0.91 (STZ vs. Control). As such, the fold changes in mt‐proteins were more dramatic in the MS performed on the subcellular fractions. Therefore, compared to the total heart, MS detection of subcellular fractions more accurately reflected changes in subcellular localized proteins under STZ induce diabetic cardiomyopathy, which might be applied to other diseases in the future.

In early 1990s, Roy et al. coined the term “metabolic memory” by demonstrating the poor reversibility of diabetic complications after normalization of glucose levels.^40^ This phenomenon deserves attention because a meta‐analysis of 37 229 diabetic patients with diabetes from eight randomized trials demonstrated that intensive glycemic control has no impact on the risk of heart failure.^41^ The mechanisms underlying this memory phenomenon are not fully understood; however, several molecular pathways, such as AGEs, oxidative stress, miRNAs, and epigenetic modifications, have been suggested to participate in its development of this phenomenon.^10^ Our study also revealed persistent dysregulation of oxidative phosphorylation and reactive oxygen species even after normalization of hyperglycemia. In contrast, other pathways, such as peroxisome, PPAR, and fatty acid metabolism, can be efficiently rescued by glycemic control. Therefore, the memory characteristics of oxidative phosphorylation and reactive oxygen species appeared to be specific. We also found that these pathways were regulated at the post‐transcriptional level, as their mRNAs levels were largely unchanged. We propose a hypothesis that there is a vicious cycle between dysregulated miRNAs and disordered mitochondrial oxidative phosphorylation. Once established, this feedback loop persisted even after the normalization of hyperglycemia in the diabetic model. Interestingly, previous studies have reported feedback loops in hyperglycemic memory. For example, ROS overproduction inhibits the activity of glyceraldehyde‐3‐phosphate dehydrogenase (GAPDH), leading to accumulation of glycolytic intermediates and increased formation of AGEs.^42^, ^43^, ^44^ AGEs promote ROS production, thereby forming a vicious cycle between ROS and AGEs.^45^, ^46^, ^47^, ^48^ Our previous study revealed that miR‐320 and CD36 mutually enhance each other's expression, leading to a positive feedback loop and a sustained hyperlipidemic state.^12^ However, the most critical initiating molecules among these feedback cycles remain to be identified. A systematic study of all the molecules/pathways involved in hyperglycemic memory using animal models at different time points might shed light on the initiating molecules.

Clinically, for diabetic patients with heart failure, blood sugar control and anti‐heart failure drugs delay disease progression. Sodium‐glucose co‐transporter‐2 inhibitors (SGLT2i) are promising therapeutic adjuvants; however, their long‐term consequences and ability to halt or even reverse disease progression remain largely unknown. Therefore, drugs that disrupt the feedback loops that contribute to hyperglycemic memory may provide new therapeutic options. Notably, a clinical trial of an antisense oligonucleotide drug in patients with HF showed that CDR132L, a specific miR‐132 inhibitor, was safe and well tolerated, confirmed linear plasma pharmacokinetics with no signs of accumulation, and suggested cardiac functional improvements.^49^ Therefore, the knockdown of upregulated miRNAs or overexpression of downregulated miRNAs may terminate the vicious cycle between miRNAs and oxidative phosphorylation. To overexpress miRNAs in cardiomyocytes, the use of rAAVs as delivery vectors is safe, as exemplified by the FDA approval of Luxturna® and Zolgensma®. Future research should focus on developing strategies that specifically overexpress downregulated miRNAs in subcellular fractions such as mitochondria.

## AUTHOR CONTRIBUTIONS

H.L. and J.Z designed the study, conducted the majority of experiments, analyzed and interpreted the data; Y.Z. and K.J. helped in acquiring the animal data; H.L., C.C. and D.W.W. wrote the paper.

## FUNDING INFORMATION

This work was supported by grants from the National Natural Science Foundation of China (nos. 82170273, Nos. 82330010, 82241034, 82270363), Tongji Hospital Science Fund for outstanding Young Scholars (no. 2020YBKY022), Hubei Key Research and Development Program (no. 2021BCA121), Knowledge Innovation Program of Wuhan‐Shuguang Project (no. 2022020801020451), and Joint Funds for the innovation of science and Technology, Fujian province (2023Y9176). The funders had no role in study design, data collection and analysis, manuscript preparation, or decision to publish.

## CONFLICT OF INTEREST STATEMENT

The authors declare that they have no competing interests.

## Supporting information


**Data S1.** Supporting information.

**Data S2.** Supporting information.

**Data S3.** Supporting information.

**Data S4.** Supporting information.

**Data S5.** Supporting information.

**Data S6.** Supporting information.

**Data S7.** Supporting information.

**Tables S1–S8.** Supporting information.

## Data Availability

Data supporting the findings of this study are available from the corresponding author upon reasonable request.

## References

[1] Saeedi P , Petersohn I , Salpea P , et al. Global and regional diabetes prevalence estimates for 2019 and projections for 2030 and 2045: results from the international diabetes federation diabetes atlas, 9(th) edition. Diabetes Res Clin Pract. 2019;157:107843. 31518657 10.1016/j.diabres.2019.107843

[2] Parim B , Sathibabu Uddandrao VV , Saravanan G . Diabetic cardiomyopathy: molecular mechanisms, detrimental effects of conventional treatment, and beneficial effects of natural therapy. Heart Fail Rev. 2019;24:279‐299. 30349977 10.1007/s10741-018-9749-1

[3] Hamby RI , Zoneraich S , Sherman L . Diabetic cardiomyopathy. JAMA. 1974;229:1749‐1754. 4278055

[4] Zinman B , Wanner C , Lachin JM , et al. Empagliflozin, cardiovascular outcomes, and mortality in type 2 diabetes. N Engl J Med. 2015;373:2117‐2128. 26378978 10.1056/NEJMoa1504720

[5] Keller DM , Ahmed N , Tariq H , et al. SGLT2 inhibitors in type 2 diabetes mellitus and heart failure‐a concise review. J Clin Med. 2022;11:11. 10.3390/jcm11061470 PMC8952302 35329796

[6] Packer M , Butler J , Filippatos GS , et al. Evaluation of the effect of sodium‐glucose co‐transporter 2 inhibition with empagliflozin on morbidity and mortality of patients with chronic heart failure and a reduced ejection fraction: rationale for and design of the EMPEROR‐reduced trial. Eur J Heart Fail. 2019;21:1270‐1278. 31584231 10.1002/ejhf.1536

[7] McMurray JJV , Solomon SD , Inzucchi SE , et al. Dapagliflozin in patients with heart failure and reduced ejection fraction. N Engl J Med. 2019;381:1995‐2008. 31535829 10.1056/NEJMoa1911303

[8] Ceriello A . Hypothesis: the “metabolic memory”, the new challenge of diabetes. Diabetes Res Clin Pract. 2009;86(Suppl 1):S2‐S6. 20115927 10.1016/S0168-8227(09)70002-6

[9] Paneni F , Volpe M , Luscher TF , Cosentino F . SIRT1, p66(Shc), and Set7/9 in vascular hyperglycemic memory: bringing all the strands together. Diabetes. 2013;62:1800‐1807. 23704521 10.2337/db12-1648 PMC3661615

[10] Zhan J , Chen C , Wang DW , Li H . Hyperglycemic memory in diabetic cardiomyopathy. Front Med. 2022;16:25‐38. 34921674 10.1007/s11684-021-0881-2

[11] Costantino S , Paneni F , Luscher TF , Cosentino F . MicroRNA profiling unveils hyperglycaemic memory in the diabetic heart. Eur Heart J. 2016;37:572‐576. 26553540 10.1093/eurheartj/ehv599

[12] Zhan J , Jin K , Ding N , et al. Positive feedback loop of miR‐320 and CD36 regulates the hyperglycemic memory‐induced diabetic diastolic cardiac dysfunction. Mol Ther Nucleic Acids. 2023;31:122‐138. 36618264 10.1016/j.omtn.2022.12.009 PMC9813582

[13] Zhan J , Lv H , Dai B , et al. The nuclear and cytoplasmic roles of miR‐320 in non‐alcoholic fatty liver disease. Aging. 2020;12:22019‐22045. 33186123 10.18632/aging.104040 PMC11623971

[14] Li H , Dai B , Fan J , et al. The different roles of miRNA‐92a‐2‐5p and let‐7b‐5p in mitochondrial translation in db/db mice. Mol Ther Nucleic Acids. 2019;17:424‐435. 31319246 10.1016/j.omtn.2019.06.013 PMC6637210

[15] Raudvere U , Kolberg L , Kuzmin I , et al. g:Profiler: a web server for functional enrichment analysis and conversions of gene lists (2019 update). Nucleic Acids Res. 2019;47:W191‐W198. 31066453 10.1093/nar/gkz369 PMC6602461

[16] Correction to: metabolic and molecular imaging of the diabetic cardiomyopathy. Circ Res. 2020;127:e78. 32614713 10.1161/RES.0000000000000416

[17] Wang L , Cai Y , Jian L , Cheung CW , Zhang L , Xia Z . Impact of peroxisome proliferator‐activated receptor‐alpha on diabetic cardiomyopathy. Cardiovasc Diabetol. 2021;20:2. 33397369 10.1186/s12933-020-01188-0 PMC7783984

[18] Tan Y , Zhang Z , Zheng C , Wintergerst KA , Keller BB , Cai L . Mechanisms of diabetic cardiomyopathy and potential therapeutic strategies: preclinical and clinical evidence. Nat Rev Cardiol. 2020;17:585‐607. 32080423 10.1038/s41569-020-0339-2 PMC7849055

[19] Karwi QG , Sun Q , Lopaschuk GD . The contribution of cardiac fatty acid oxidation to diabetic cardiomyopathy severity. Cells. 2021;10:3259. 10.3390/cells10113259 PMC8621814 34831481

[20] Ouwens DM , Diamant M , Fodor M , et al. Cardiac contractile dysfunction in insulin‐resistant rats fed a high‐fat diet is associated with elevated CD36‐mediated fatty acid uptake and esterification. Diabetologia. 2007;50:1938‐1948. 17639306 10.1007/s00125-007-0735-8 PMC2039861

[21] Lee TW , Bai KJ , Lee TI , Chao TF , Kao YH , Chen YJ . PPARs modulate cardiac metabolism and mitochondrial function in diabetes. J Biomed Sci. 2017;24:5. 28069019 10.1186/s12929-016-0309-5 PMC5223385

[22] Day SM , Tardiff JC , Ostap EM . Myosin modulators: emerging approaches for the treatment of cardiomyopathies and heart failure. J Clin Invest. 2022;132:e148557. 10.1172/JCI148557 PMC8884898 35229734

[23] Chen D , Ruan X , Liu Y , He Y . HMGCS2 silencing attenuates high glucose‐induced in vitro diabetic cardiomyopathy by increasing cell viability, and inhibiting apoptosis, inflammation, and oxidative stress. Bioengineered. 2022;13:11417‐11429. 35506308 10.1080/21655979.2022.2063222 PMC9275940

[24] Uchihashi M , Hoshino A , Okawa Y , et al. Cardiac‐specific Bdh1 overexpression ameliorates oxidative stress and cardiac remodeling in pressure overload‐induced heart failure. Circ Heart Fail. 2017;10:e004417. 10.1161/CIRCHEARTFAILURE.117.004417 29242353

[25] Tan W , Bao H , Liu Z , Liu Y , Hong L , Shao L . PDK4 protein interacts with Hmgcs2 to facilitate high glucose‐induced myocardial injuries. Curr Mol Med. 2022;23:1104‐1115. 10.2174/1566524023666221021124202 36281857

[26] Zhao G , Jeoung NH , Burgess SC , et al. Overexpression of pyruvate dehydrogenase kinase 4 in heart perturbs metabolism and exacerbates calcineurin‐induced cardiomyopathy. Am J Physiol Heart Circ Physiol. 2008;294:H936‐H943. 18083902 10.1152/ajpheart.00870.2007

[27] Li H , Fan J , Zhao Y , et al. Nuclear miR‐320 mediates diabetes‐induced cardiac dysfunction by activating transcription of fatty acid metabolic genes to cause lipotoxicity in the heart. Circ Res. 2019;125:1106‐1120. 31638474 10.1161/CIRCRESAHA.119.314898 PMC6903355

[28] Fabian MR , Sonenberg N . The mechanics of miRNA‐mediated gene silencing: a look under the hood of miRISC. Nat Struct Mol Biol. 2012;19:586‐593. 22664986 10.1038/nsmb.2296

[29] Gebert LFR , MacRae IJ . Regulation of microRNA function in animals. Nat Rev Mol Cell Biol. 2019;20:21‐37. 30108335 10.1038/s41580-018-0045-7 PMC6546304

[30] Valinezhad Orang A , Safaralizadeh R , Kazemzadeh‐Bavili M . Mechanisms of miRNA‐mediated gene regulation from common downregulation to mRNA‐specific upregulation. Int J Genomics. 2014;2014:970607. 25180174 10.1155/2014/970607 PMC4142390

[31] Zhang X , Zuo X , Yang B , et al. MicroRNA directly enhances mitochondrial translation during muscle differentiation. Cell. 2014;158:607‐619. 25083871 10.1016/j.cell.2014.05.047 PMC4119298

[32] Zhao X , Wang Q , Lin F , et al. RNA sequencing of osteosarcoma gene expression profile revealed that miR‐214‐3p facilitates osteosarcoma cell proliferation via targeting Ubiquinol‐cytochrome c reductase Core protein 1 (UQCRC1). Med Sci Monit. 2019;25:4982‐4991. 31276465 10.12659/MSM.917375 PMC6626500

[33] Wang J , Jia Z , Zhang C , et al. miR‐499 protects cardiomyocytes from H_2_O_2_‐induced apoptosis via its effects on Pdcd4 and Pacs2. RNA Biol. 2014;11:339‐350. 24646523 10.4161/rna.28300 PMC4075519

[34] Lv G , Shao S , Dong H , Bian X , Yang X , Dong S . MicroRNA‐214 protects cardiac myocytes against H_2_O_2_‐induced injury. J Cell Biochem. 2014;115:93‐101. 23904244 10.1002/jcb.24636

[35] Meng Q , Liu Y , Huo X , Sun H , Wang Y , Bu F . MicroRNA‐221‐3p contributes to cardiomyocyte injury in H_2_O_2_‐treated H9c2 cells and a rat model of myocardial ischemia‐reperfusion by targeting p57. Int J Mol Med. 2018;42:589‐596. 29693157 10.3892/ijmm.2018.3628

[36] Yu Y , Liu H , Yang D , et al. Aloe‐emodin attenuates myocardial infarction and apoptosis via up‐regulating miR‐133 expression. Pharmacol Res. 2019;146:104315. 31207343 10.1016/j.phrs.2019.104315

[37] Pappireddi N , Martin L , Wuhr M . A review on quantitative multiplexed proteomics. Chembiochem. 2019;20:1210‐1224. 30609196 10.1002/cbic.201800650 PMC6520187

[38] Prasad B , Achour B , Artursson P , et al. Toward a consensus on applying quantitative liquid chromatography‐tandem mass spectrometry proteomics in translational pharmacology research: a white paper. Clin Pharmacol Ther. 2019;106:525‐543. 31175671 10.1002/cpt.1537 PMC6692196

[39] Vidova V , Spacil Z . A review on mass spectrometry‐based quantitative proteomics: targeted and data independent acquisition. Anal Chim Acta. 2017;964:7‐23. 28351641 10.1016/j.aca.2017.01.059

[40] Roy S , Sala R , Cagliero E , Lorenzi M . Overexpression of fibronectin induced by diabetes or high glucose: phenomenon with a memory. Proc Natl Acad Sci U S A. 1990;87:404‐408. 2296596 10.1073/pnas.87.1.404 PMC53272

[41] Castagno D , Baird‐Gunning J , Jhund PS , et al. Intensive glycemic control has no impact on the risk of heart failure in type 2 diabetic patients: evidence from a 37,229 patient meta‐analysis. Am Heart J. 2011;162:938‐948.e2. 22093212 10.1016/j.ahj.2011.07.030

[42] Bianchi C , Miccoli R , Del Prato S . Hyperglycemia and vascular metabolic memory: truth or fiction? Curr Diab Rep. 2013;13:403‐410. 23456482 10.1007/s11892-013-0371-2

[43] Cooper ME , El‐Osta A . Epigenetics: mechanisms and implications for diabetic complications. Circ Res. 2010;107:1403‐1413. 21148447 10.1161/CIRCRESAHA.110.223552

[44] Du X , Matsumura T , Edelstein D , et al. Inhibition of GAPDH activity by poly(ADP‐ribose) polymerase activates three major pathways of hyperglycemic damage in endothelial cells. J Clin Invest. 2003;112:1049‐1057. 14523042 10.1172/JCI18127 PMC198524

[45] Ko SY , Ko HA , Chu KH , et al. The possible mechanism of advanced glycation end products (AGEs) for Alzheimer's disease. PLoS One. 2015;10:e0143345. 26587989 10.1371/journal.pone.0143345 PMC4654523

[46] Chen YH , Chen ZW , Li HM , Yan XF , Feng B . AGE/RAGE‐induced EMP release via the NOX‐derived ROS pathway. J Diabetes Res. 2018;2018:6823058. 29744367 10.1155/2018/6823058 PMC5878883

[47] Wautier MP , Chappey O , Corda S , Stern DM , Schmidt AM , Wautier JL . Activation of NADPH oxidase by AGE links oxidant stress to altered gene expression via RAGE. Am J Physiol Endocrinol Metab. 2001;280:E685‐E694. 11287350 10.1152/ajpendo.2001.280.5.E685

[48] Cepas V , Collino M , Mayo JC , Sainz RM . Redox signaling and advanced glycation endproducts (AGEs) in diet‐related diseases. Antioxidants. 2020;9:142. 10.3390/antiox9020142 PMC7070562 32041293

[49] Taubel J , Hauke W , Rump S , et al. Novel antisense therapy targeting microRNA‐132 in patients with heart failure: results of a first‐in‐human phase 1b randomized, double‐blind, placebo‐controlled study. Eur Heart J. 2021;42:178‐188. 33245749 10.1093/eurheartj/ehaa898 PMC7954267

