# Supplementary material for: Subcellular mass spectrometric detection unveils hyperglycemic memory in the diabetic heart
Source: J Diabetes. 2024 Nov 13;16(11):e70033. doi: 10.1111/1753-0407.70033 (PMC11561303; doi:10.1111/1753-0407.70033)
Supplement: Supplementary file 8 — Tables S1–S8. Supporting information. [file JDB-16-e70033-s002.docx]

**Supplementary Table 1. Antibodies used in the present study.**

| Antibody | Company | Catalog number | Final antibody dilutions |
| --- | --- | --- | --- |
| GAPDH | Proteintech | 60004-1-Ig | 1:5000 |
| HMGCS2 | Abcam | Ab137043 | 1:2000 |
| MYH7 | Proteintech | 22280-1-AP | 1:2000 |
| PDK4 | Abclonal | A13337 | 1:2000 |
| BDH1 | Proteintech | 15417-1-AP | 1:5000 |

**Supplementary Table 2. Comparison information about glycemic control in present study and the previous study containing miRNA-seq data.**

|  | Present study | Previous study  (doi:10.1093/eurheartj/ehv599) |
| --- | --- | --- |
| Initiation time of insulin treatment | After 4 weeks of hyperglycemia | After 3 weeks of hyperglycemia |
| Duration of insulin treatment | 4 weeks | 3 weeks |
| Insulin treatment method | Osmotic pump (Durect, Cupertino, CA, USA) | Insulin implant (LinBit, LinShin, Canada) |
| Insulin type | Insulin aspart | - |
| Glycemic levels reached | Revert to the control group level | Revert to the control group level |

Previous study referred to the study from where the miRNA-seq data were obtained.
